# Agomelatine rescues lipopolysaccharide-induced neural injury and depression-like behaviors via suppression of the Gαi-2-PKA-ASK1 signaling pathway

**DOI:** 10.1186/s12974-022-02479-x

**Published:** 2022-05-24

**Authors:** Tian Lan, Yuhan Wu, Yulei Zhang, Shuhan Li, Zhanpeng Zhu, Liyan Wang, Xueqin Mao, Ye Li, Cuiqin Fan, Wenjing Wang, Shu Yan Yu

**Affiliations:** 1 Department of Physiology, School of Basic Medical Sciences, Shandong University, 44 Wenhuaxilu Road, Jinan, Shandong Province 250012 People’s Republic of China; 2 Jinan International Travel Healthcare Center, Wenhuadonglu Road 62#, Jinan, Shandong Province 250012 People’s Republic of China; 3 Morphological Experimental Center, School of Basic Medical Sciences, Shandong University, 44 Wenhuaxilu Road, Jinan, Shandong Province 250012 People’s Republic of China; 4 Department of Psychology, Qilu Hospital of Shandong University, 107 Wenhuaxilu Road, Jinan, Shandong Province 250012 People’s Republic of China; 5 Shandong Provincial Key Laboratory of Mental Disorders, School of Basic Medical Sciences, 44 Wenhuaxilu Road, Jinan, Shandong Province 250012 People’s Republic of China

**Keywords:** Agomelatine, 5-HT2C receptor, Gαi-2, Apoptosis, Depression

## Abstract

**Background:**

Agomelatine has been shown to be effective in the treatment of depression, but the molecular mechanisms underlying its antidepressant effects have yet to be elucidated. Identification of these molecular mechanisms would not only offer new insights into the basis for depression but also provide the foundation for the development of novel treatments for this disorder.

**Methods:**

Intraperitoneal injection of LPS was used to induce depression-like behaviors in rats. The interactions of the 5-HT2C reporter and Gαi-2 were verified by immunoprecipitation or immunofluorescence assay. Inflammatory related proteins, autophagy related proteins and apoptosis markers were verified by immunoblotting or immunofluorescence assay. Finally, electron microscopy analysis was used to observe the synapse and ultrastructural pathology.

**Results:**

Here, we found that the capacity for agomelatine to ameliorate depression and anxiety in a lipopolysaccharide (LPS)-induced rat model of depression was associated with an alleviation of neuroinflammation, abnormal autophagy and neuronal apoptosis as well as the promotion of neurogenesis in the hippocampal dentate gyrus (DG) region of these rats. We also found that the 5-HT2C receptor is coupled with G alphai (2) (Gαi-2) protein within hippocampal neurons and, agomelatine, acting as a 5-HT2C receptor antagonist, can up-regulate activity of the Gαi-2-cAMP-PKA pathway. Such events then suppress activation of the apoptosis signal-regulating kinase 1 (ASK1) pathway, a member of the mitogen-activated protein kinase (MAPK) family involved in pathological processes of many diseases.

**Conclusion:**

Taken together, these results suggest that agomelatine plays a neuroprotective role in regulating neuroinflammation, autophagy disorder and apoptosis in this LPS-induced rat model of depression, effects which are associated with the display of antidepressant behaviors. These findings provide evidence for some of the potential mechanisms for the antidepressant effects of agomelatine.

**Graphical Abstract:**

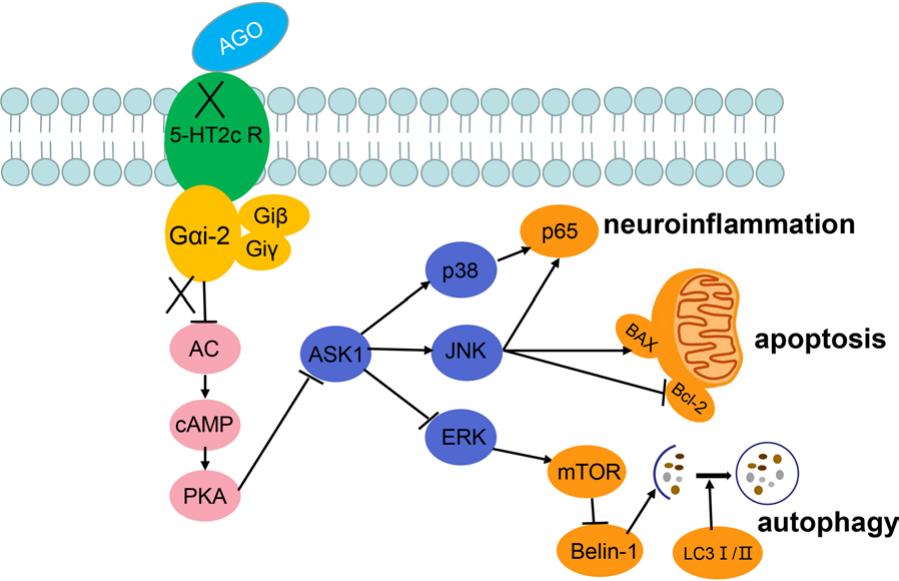

**Supplementary Information:**

The online version contains supplementary material available at 10.1186/s12974-022-02479-x.

## Highlights

1. Agomelatine ameliorated anxiety and depression-like behaviors in rats.
2. Agomelatine regulated LPS-induced neuroinflammation and neuronal apoptosis.
3. Agomelatine decreased the enhanced autophagy and synaptic damage in LPS-treated rats.
4. Agomelatine alleviated neural injury via suppression of Gαi-2-PKA-ASK1 pathway.

**Supplementary Information:**

The online version contains supplementary material available at 10.1186/s12974-022-02479-x.

## Background

Depression is considered a psychiatric illness usually resulting from external stress stimulation [1]. Pathologically, depression is characterized by alterations in neuronal structures and/or functional injuries, mainly localized within specific brain regions [2, 3]. Neuroinflammation, abnormal autophagy and apoptosis are prerequisites for neuronal injury, and also represent an important basis for the onset and development of cognitive dysfunction in patients with depression [4–6]. Therefore, prevention of the neuronal injury and/or promotion of neuronal repair within these specific regions have important clinical implications for the treatment of depression [7].

Inflammation is triggered by any injury or infection to cells and tissues, leading to various biochemical cascades that aimed to address these threats to homeostasis [8]. In the brain, neuroinflammation is manifested by increased levels of pro-inflammatory cytokines, microglia activation and nerve tissue damage. During neuroinflammation, immunogenic molecules can activate microglia and greatly increase the production of cytokines and reactive oxygen species (ROS) [9]. At the same time, under conditions of continuous high intensity stimulation or severe neuroinflammation, autophagic mechanisms are disrupted as a result of changes in expressions of the autophagic specific proteins Beclin-1, LC3 and P62 [10]. Moreover, dysfunctional autophagic mechanisms may further activate apoptotic signal pathways in the middle and downstream components of neuronal pathways, eventually leading to neuronal death and/or activation of glial cells, which further exacerbates neuroinflammation and leads to decreased synaptic plasticity and compromised learning and memory [11–13]. Neuroinflammation has also been shown to be present in most central nervous system diseases, and over the past few decades there has been much evidence that systemic inflammation can lead to depressive-like behavior in rodents and depressive symptoms in some healthy volunteers [14]. Although it is not entirely certain that all cases of depression are associated with inflammation, it suggests that inflammation-related depression, which is common in treatment-resistant depression, should be considered as a type of major depression, and that the amelioration of neuroinflammation is undoubtedly one of the ways to better search for more effective treatments.

One new drug that has been shown to be efficacious in the treatment of severe depression is agomelatine, which acts as an agonist of melatonin MT1 and MT2 receptor and a neutral antagonist of serotonin receptor (5-HT2C) [15–17]. Among the common antidepressants, agomelatine have a lower risk of triggering withdrawal syndrome and is well tolerated and safe [18, 19]. Agomelatine has also been shown to increase hippocampal neurogenesis, enhance the expression of brain-derived neurotrophic factor (BDNF) and play a protective role in oxidative damage and neuroinflammation in the brain [20–22]. Meanwhile, agomelatine also exerts protective role in the treatment and prevention of cardiovascular diseases, type 2 diabetes and other diseases [23, 24].

However, details regarding the potential molecular mechanisms for these antidepressant effects of agomelatine remain unclear, and the mechanisms of agomelatine regulates neuronal intracellular signaling by antagonizing 5-HT2C receptor also remain largely unexplored. Therefore, in this study, we investigated some of the possible mechanisms by which agomelatine exerts neuroprotective effects in a rat model of depression as resulting from increased neuroinflammation induced by LPS. We found that agomelatine affects the inhibitory guanine nucleotide regulatory protein Gαi-2 and downstream intracellular molecules possibly through antagonistic actions upon 5-HT2C receptors. Such effects exert beneficial effects through the capacity to improve inflammation-induced immune changes, hippocampal autophagy and neuronal apoptosis.

## Methods

### Animals and housing conditions

Male Wistar rats weighing 200–220 g (~ 9 weeks of age) were purchased from the Experimental Animal Centre of Shandong University. All experiments were approved by the Shandong University Animal Care and Use Committee and were conducted according to the International Guiding Principles for Animal Research provided by the International Organizations of the Medical Sciences Council. Rats were housed in CP-4 cages under a 12 h light/dark cycle and had free access to food and water, the cages were cleaned and disinfected twice a week. Ambient temperature was maintained at 22 °C ± 2 °C and humidity (55 ± 5)%, except when subjected to conditions of specific experiments. All efforts were made to minimize the pain and numbers of the animals used in the experiments.

### Regents and antibodies

Lipopolysaccharide (LPS) from Escherichia coli O55:B5 was purchased from Sigma-Aldrich (St Louis, MO, USA) and agomelatine from Aladdin (Shanghai, China). The monoclonal rabbit anti-LC3 (12741), anti-mTOR (2972), anti-Beclin-1 (3495), anti-p62 (23214), anti-ERK (9102), anti‐phospho‐JNK (4668), anti‐phospho‐ASK1 (3764), anti‐phospho‐p38 (9211), anti-Cleaved Caspase3 (9661), anti-Doublecortin (4604) and anti-β-actin (4970) were all purchased from Cell Signaling Technology. Anti-Gαi-2 (sc-13534) was purchased from Santa Cruz Biotechnology, anti-NF-κB P65 (bs90940), anti-phospho-PKA (bs4345) from Bioworld and anti-5-HT2C Receptor (DF3501) from Affinity Biosciences. Polyclonal rabbit anti-ionized calcium binding adaptor molecule-1 (Iba-1) (019-19741) was purchased from Wako Pure Chemical Inc. and Hoechst 33258 (C0031) from Solarbio. The polyclonal goat anti‐rabbit secondary antibody and the polyclonal goat anti‐mouse secondary antibody were purchased from Beijing Zhongshan Golden Bridge. Alexa-568 goat anti-rabbit IgG secondary antibody was purchased from Invitrogen and Alexa-488 goat anti-mouse IgG secondary antibody from Abcam Plc.

### Animal model of depression

The LPS-induced rat model of depression used was that as described in the previous literature with minor modifications [25]. Briefly, rats received a daily intraperitoneal (i.p.) injection of LPS (0.5 mg/kg) for 10 consecutive days. Solutions were prepared immediately prior to injection. Identical schedules of equal amounts of saline were administered to rats serving as controls.

### Drug treatments

Agomelatine was dissolved in 1% hydroxyethyl cellulose (HEC) solution and LPS in 0.9% saline. In all experiments, agomelatine (40 mg/kg) was administered via an i.p. injection at 60 min prior to daily LPS procedures. All rats received a daily i.p. injection of saline (10 ml/kg) for three days prior to the experiment for habituation and were then randomly allocated to one of the following groups with *N* = 30/group: (a) control (non-stressed group), (b) LPS (0.5 mg/kg daily), (c) LPS treated with agomelatine (LPS + agomelatine) and (d) LPS treated with 1% HEC solution (LPS + HEC). The dose and intraperitoneal injection regimen of agomelatine administration was based upon results of a previous study [21, 26].

### Behavioral tests

In order to verify the effects of agomelatine treatment and LPS injection on depressive-like behavior, four sets of animals were tested 24 h after the last LPS injection. All behavioral experiments were conducted in isolated behavioral testing rooms and performed by experimenters who were blind as to the identity of the experimental groups.

### Sucrose preference test

The sucrose preference test (SPT), which serves as a means of assessing anhedonia responses in rats. The SPT was conducted as described previously with minor modifications [27]. Briefly, rats were placed individually in cages with two bottles of sucrose solution (1%, w/v) for an initial 24 h period followed by replacement of one of the bottles of sucrose solution with tap water for the second 24 h period. Familiarization phase parallel with animal modeling period. After this adaptation phase, rats were deprived of food and water for 24 h and then permitted access to two bottles for 3 h, one bottle containing 100 ml of 1% sucrose solution and the other containing 100 ml of tap water. The test phase of the experiment started 24 h after the last LPS injection and familiarization phase. Sucrose preference was defined as sucrose consumption/[water consumption + sucrose consumption] × 100% during the test phase.

### Forced swimming test

The forced swimming test (FST) was used to evaluate behavioral despair in rats. After 10 days of LPS treatment, rats were placed individually in a water filled cylinder (height: 80 cm, diameter: 30 cm, 25 °C) for 15 min of forced swimming in the training session. At 24 h after this training session, the rats were again placed in the water filled cylinder for a 5 min test session. During this 5 min test session, immobility and horizontal movement times were recorded. Rats floating motionless without struggling or making only minimal movements necessary to maintain their heads above the water surface were considered as immobile. Horizontal movement throughout the cylinder was defined as swimming while vertical movement against the wall of the cylinder was defined as climbing.

### The elevated plus maze test

The elevated plus maze (EPM) was used as a means to assess anxiety in rats. The EPM consists of two closed arms, two open arms (50 cm × 10 cm) and a central platform (10 cm × 10 cm) at the intersection of the four arms. The apparatus is suspended at > 50 cm above the ground surface. During the 5-min test, the animal was first placed on the central platform with their heads oriented toward the open arms. The time spent in the open arms and number of entries into the open arms in the 5 min test were recorded using the Smart software (SMART is a video tracking platform software).

### Open field test

The open field test was used to evaluate autonomous movement and anxiety-like behavior. The experimental device is a square plastic box (150 × 150 × 50 cm^3^). All the rats were placed in the center of the open field apparatus, allowed them to explore freely for 5 min. The total distance and the time spent in the central area within 5 min were recorded by the video tracking software (SMART 2.5, Spain). After each rat has been tested, the box was swabbed with a wet cloth to prevent the smell from interfering with the next test.

### Brain anatomy and tissue preparation

Twenty-four hours after completion of behavioral tests, rats from each group were anesthetized with sodium phenobarbital (30 mg/kg) and perfused transcardially with 300 ml 0.9% NaCl containing heparin sodium salt followed by fixation with 4% paraformaldehyde (PFA). Brains were removed and post-fixed in 4% PFA overnight at 4 °C followed by a graded dehydration. Brain tissue was encased at an optimum cutting temperature compound and frozen serial coronal sections were cut. Hippocampal sections were selected for immunofluorescent staining.

### Immunofluorescent staining and confocal microscopy

Frozen slices (40 μm) were incubated overnight at 4 °C with the specific primary antibodies. Sections were then incubated with the fluorescent-conjugated secondary antibody for 1 h at 37℃ in a thermostatic oscillator. DAPI (Beyotime) was used for nuclear staining. Before each step, slices were washed three times with PBS. Images were captured with use of a confocal microscope (LSM880, Carl Zeiss, Germany) and processed using ZEN software. The fluorescent intensity analysis was performed using Image J (NIH, Bethesda, MD, USA). Positive cells of Iba1 per 1 mm^2^ were counted by investigators. All experiments were conducted in a blinded manner, adhering to stereological principles.

### Western blotting and immunoprecipitation

Rats were anesthetized with an intraperitoneal injection of sodium phenobarbital (30 mg/kg) as based on their body weight. Within the hippocampus, the CA1 region was located on the dorsal side of the brain and the DG was located on the ventral side of the brain. Under the stereomicroscope, the CA1 and DG regions were divided along the bisected hippocampal fissure on the ventral surface of the hippocampus. Bilateral DG tissue samples were isolated and lysed in RIPA buffer containing a cocktail of protease/phosphatase inhibitors. After centrifugation (20 min, 12,000 rpm, 4 °C), cleared lysates containing the isolated proteins were harvested. The membrane protein was extracted using the Membrane and Cytosol Protein Extraction Kit (Beyotime P0033). Protein concentrations were determined using the Pierce BCA protein kit. Western blot was performed as described previously [28]. Proteins (30 μg) from each tissue sample were loaded in each lane, electrophoretically separated on SDS-PAGE gels and then transferred onto PVDF membranes for primary antibody incubation at 4 °C overnight. The polyclonal goat anti‐rabbit secondary antibody and polyclonal goat anti‐mouse secondary antibody were used as labelled secondary antibodies to detect the proteins of interest. Image-J software was used to perform pixel quantifications of the images. Intra-run normalization against the internal actin control was performed for each sample. For immunoprecipitation (IP), tissue samples were lysed in NP-40 lysis buffer (Beyotime, Haimen, China) and a protease inhibitor cocktail (Merck). After centrifugation for 15 min at 12,000 rpm at 4 °C, supernatants were collected and incubated with specific antibody at 4 °C for 6 h. Protein A + G agarose IP (Biowold, BD0048) were then added to the mix and rotated overnight at 4 °C. After incubation, bead‐linked immune complexes were washed with IP buffer 5 times. Immunoprecipitation was performed by boiling with 1% SDS buffer and analyzed by Western blot.

### Real-time quantitative PCR

According to the manufacturers’ instructions, total RNA was isolated from hippocampal DG regions using the Trizol kit (Invitrogen) and was reverse transcribed into cDNA using reverse transcriptase (GeneCopoeia, USA). Quantitative real-time expression levels of mRNA were determined using the mRNA qRT-PCR detection kit (GeneCopoeia, USA). Real-time quantitative PCR analysis was performed on a Bio-Rad iCycler system (Bio-Rad, Hercules, CA). GAPDH served as a loading control for the samples to test for mRNA, with mRNA expression levels evaluated using the 2 − (^ΔΔ^Ct) method. Sequences of specific primers are listed in Additional file 4: Table S1.

### Transmission electron microscopy (TEM)

The ultrastructure of DG neurons was observed with use of TEM (Philips Tecnai 20 U-Twin, Holland). Bilateral DG tissues were carefully dissected (1 × 1 × 1 mm), fixed with 1% osmium tetroxide for 2 h, and dehydrated with graded ethanol. The tissue was infiltrated overnight with a semi-epoxy-propane mixture and then embedded in resin. Tissues were cut into ultrathin section (70 nm), stained with 4% uranyl acetate for 20 min and then stained with 0.5% lead citrate on the copper grid. At least 30 micrographs were randomly selected from each rat and analyzed using Image J analysis software (NIH, Scion Corporation, Frederick, MD).

### Enzyme-linked immunosorbent assay (ELISA)

Concentrations of cyclic adenosine monophosphate (cAMP) were determined using an ELISA kit (Enzyme-linked Biotechnology Co. Shanghai, China) according to the manufacturers’ instructions. Equal amounts of diluted samples were added to 96-well plates. The data were expressed as the amount of cAMP (pg) per total protein (mg) (mean ± SEM) with duplicate detections performed a minimum of three times. The statistical results were expressed as percentage ratios relative to control group.

### Statistical analysis

All calculations were performed using GraphPad Prism 8.0.1 (GraphPad Software, Inc., San Diego, CA). All data are presented as the means ± standard errors of the mean. Statistical significance of differences between the groups was evaluated with use of two-way analysis of variance (ANOVA), with the Tukey’s multiple-comparison test used for post hoc comparisons. A value of *P* < 0.05 was required for the results to be considered statistically significant.

## Results

### Agomelatine treatment reverses depression-like behaviors in LPS rats

**Fig. 1:**
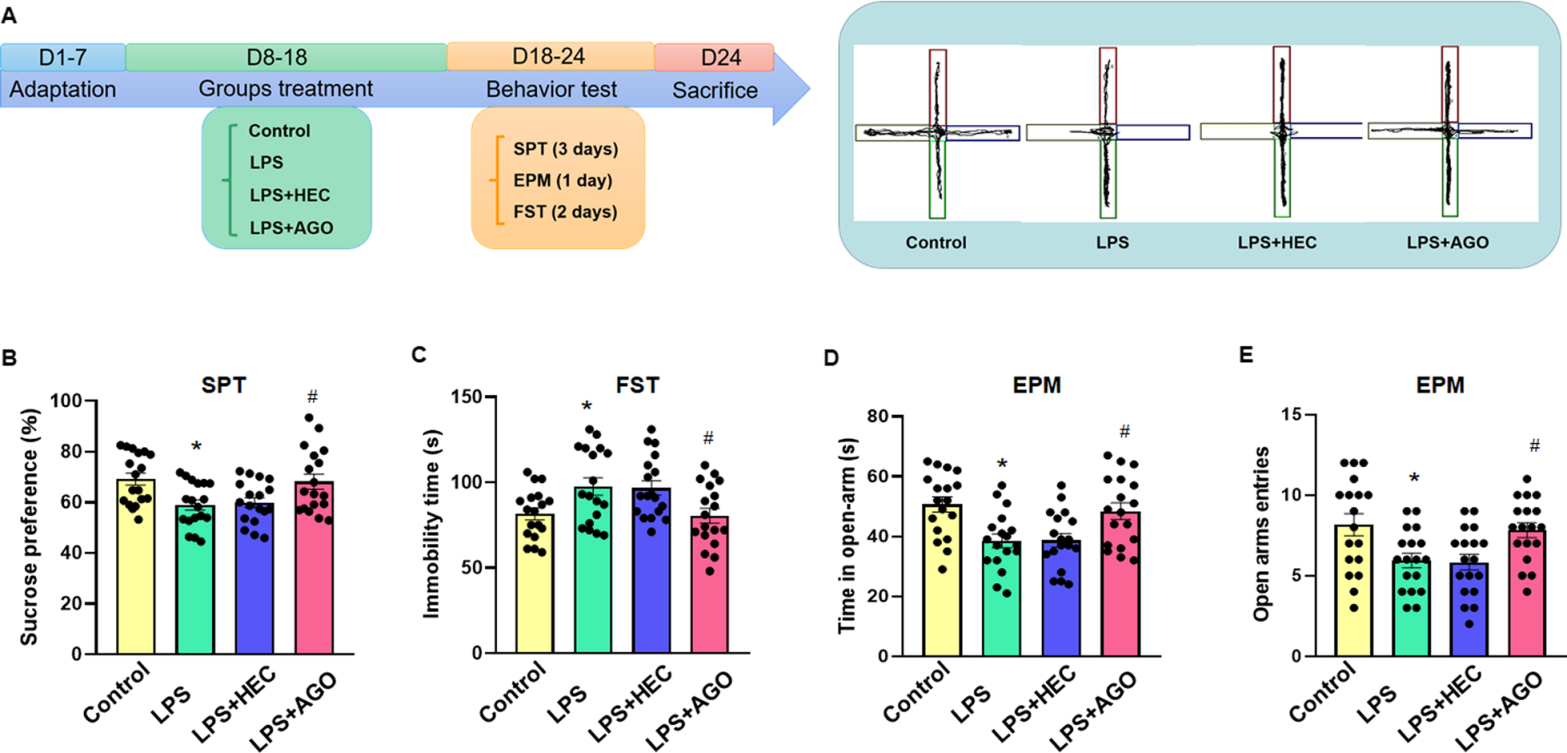
Agomelatine reverses LPS-induced depression-like behaviors in rats. **A** Schematic diagram of experimental design. **B** Agomelatine reversed the decreases in percent of sucrose consumption in the SPT and **C** increased immobility times in the FST in the LPS-induced rat model of depression. **D** Amount of time in the open arms and **E** number of entries into the open arms in the EPM test. *N* = 18 per group, *P < 0.05, LPS vs Control group; ^#^*P* < 0.05, LPS vs LPS + AGO (AGO, Agomelatine). The data are presented as means ± SEMs

In order to verify that this LPS-induced model of depression was effective in producing behavioral indices of depression and that agomelatine can improve these displays of depression, responses in three different behavioral tests were assessed (Fig. 1A). LPS treatment decreased the percent of sucrose solution consumed in these rats (*F*_(3,51)_ = 4.762, *P* < 0.01, Fig. 1B) and increased immobility times in the FST (*F*_(3,51)_ = 5.216, *P* < 0.01, Fig. 1C). In the EPM test, we primarily examined anxiety-like behavior in rats, the time (*F*_(3,51)_ = 5.801, *P* < 0.01) and number of entries into the open arms (*F*_(3,51)_ = 4.749, *P* < 0.01) were also significantly reduced in LPS rats (Fig. 1D, E). The OFT was used to measure autonomous movement and exploratory behavior in new environments (Additional file 2: Figure S2). The LPS treatment resulted in a decrease in the time spent in the central area compared with the control group (F _(3, 15)_ = 7.990, *P* < 0.01). The significant reduction in spontaneous movement was not observed in any of the groups (*F*_(3,15)_ = 0.3225, *P* > 0.05). All these behavioral responses following LPS treatment were essentially reversed in LPS rats receiving agomelatine. At the same time, we also demonstrated that agomelatine did not increase non-specifically general activity in normal rats (Additional file 3: Fig. S3).

### The antidepressant effects of agomelatine appear to involve a regulation of downstream pathways through interactions of the 5-HT2C reporter and Gαi-2

**Fig. 2:**
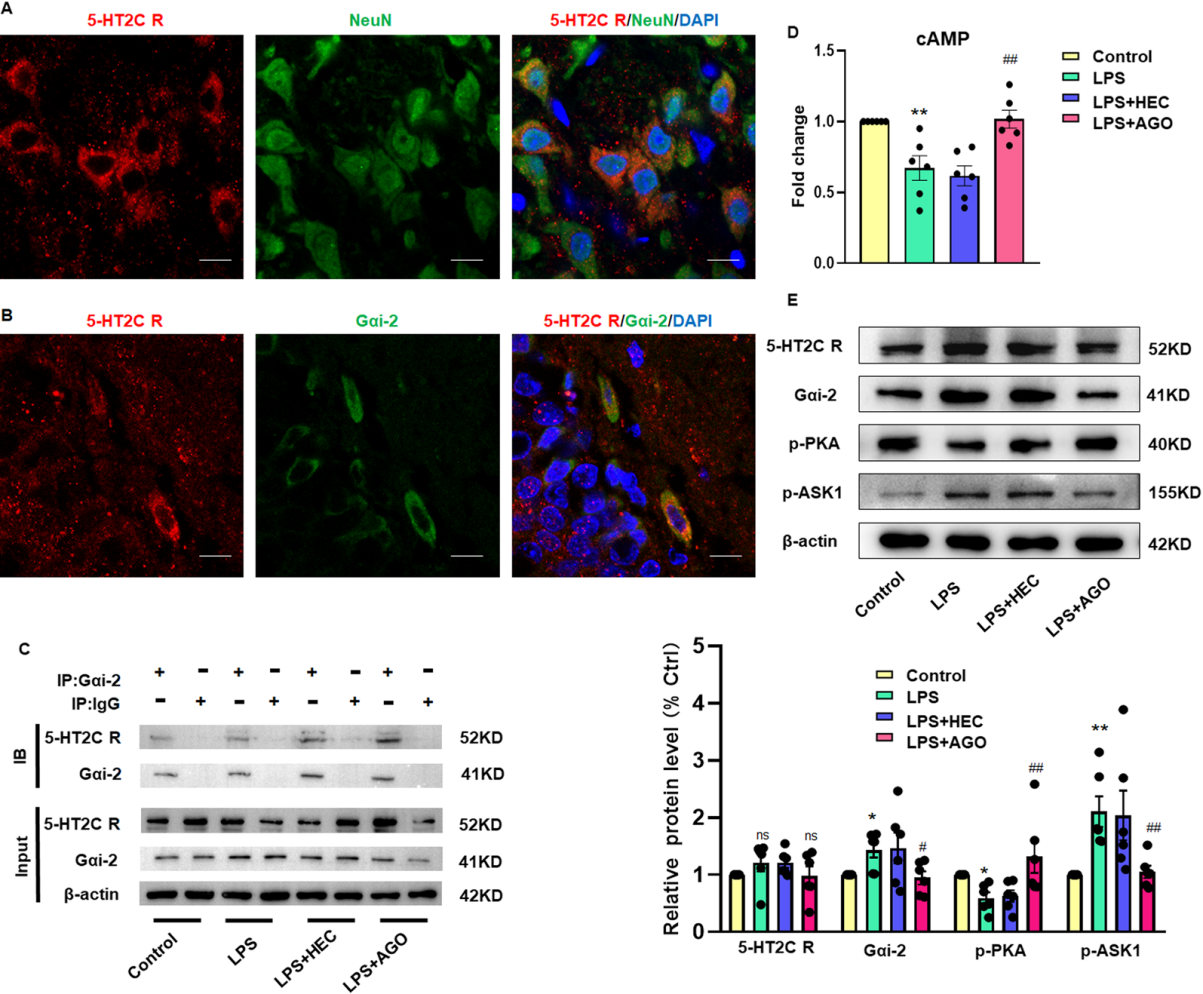
Agomelatine appears to exert antidepressant effects through regulation of downstream pathways involving interactions of the 5-HT2C reporter and Gαi-2. **A** Representative images of the 5-HT2C receptor (red). Nuclei (blue) are stained with DAPI. Neurons (green) are stained with Anti-NeuN. Scale bar is 5 μm (*N* = 6 per group). **B** Representative images of laser scanning confocal microscopy of the 5-HT2C receptor (red) and Gαi-2 (green) in the hippocampal DG region. **C** CO-IP analysis of endogenous interactions between the 5-HT2C receptor and Gαi-2 in the DG region. **D** ELISA analysis of cAMP in the DG region of LPS-treated rats receiving a pretreatment of agomelatine. **E** Western blot assays of protein expression levels of the 5-HT2C receptor, Gαi-2, P-PKA and P-ASK1within the DG region (*N* = 6 per group). *P < 0.05, **P < 0.01, LPS vs Control group; ^#^*P* < 0.05, ^##^*P* < 0.01, LPS vs LPS + AGO (AGO Agomelatine). Data are presented as means ± SEMs

As an approach to assess these interactions of the 5-HT2C reporter and Gαi-2, we first verified the presence of 5-HT2C receptors in neurons within the hippocampal DG region of rats (Fig. 2A). Then, through immunofluorescent co-localization and immunoprecipitation experiments, we demonstrated that the 5-HT2C receptor was coupled with Gαi-2 (Fig. 2B, C). As shown in Fig. 2E, agomelatine inhibited 5-HT2C receptor activation, but the total protein expression of 5-HT2C receptor and its protein expression on the cell membrane (Additional file 1: Fig. S1) were not significantly altered in response to treatment with agomelatine. However, in the LPS-induced model of depression, activation of the 5-HT2C receptor (*F*_(3,15)_ = 1.680, *P* > 0.05) as coupled with Gαi-2 (*F*_(3,15)_ = 6.931, *P* < 0.01) mediated the decreases in downstream cAMP (*F*_(3,15)_ = 14.31, *P* < 0.001, Fig. 2D) and PKA (*F*_(3,15)_ = 8.419, *P* < 0.01) expression and increased ASK1 (*F*_(3,15)_ = 9.437, *P* < 0.001). In antibody capture/scintillation proximity assays, agomelatine concentration dependently and competitively abolished 5-HT2C receptor-mediated activation of Gq/11 and Gαi-3 [29]. As shown in Fig. 2D, the increase of intracellular cAMP content after agomelatine treatment also indirectly confirmed that the antagonistic effect of agomelatine inhibited Gαi-2 conjugated with 5-HT2C receptor. Agomelatine appears to decrease the expression of Gαi-2 via antagonism of the 5-HT2C receptor; thereby, reducing ASK1 and thus producing antidepressant effects.

### Agomelatine alleviates neuronal inflammation in the DG region of LPS-induced rats

**Fig. 3:**
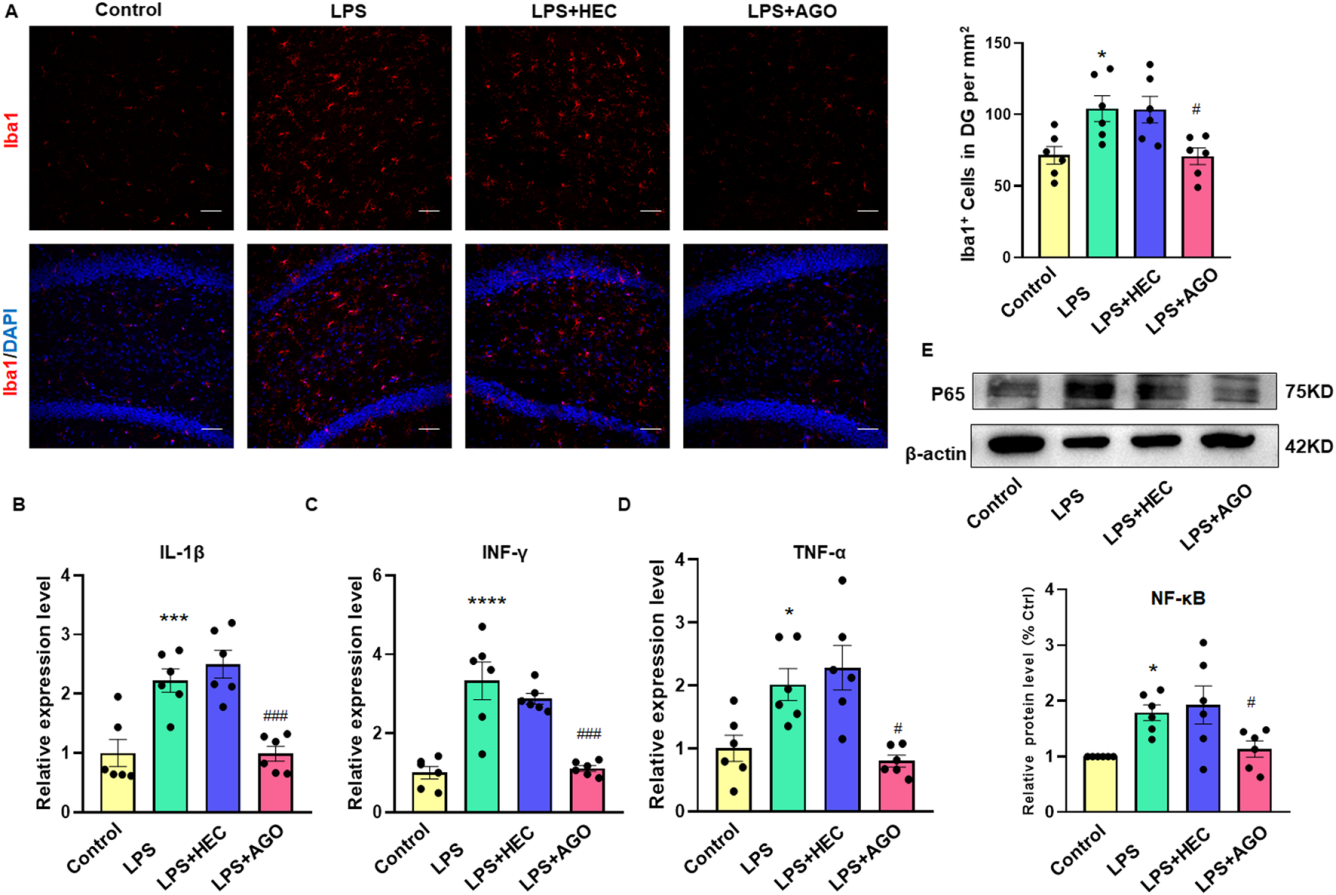
Agomelatine alleviates neuronal inflammation in the DG region of LPS rats. **A** Immunofluorescent staining of Iba1 positive microglial cells within the DG region. Scale bar is 50 μm. (*N* = 6 per group). **B**–**D** Q-PCR showing mRNA levels of pro-inflammatory cytokines. Band intensities were normalized to GAPDH. (*N* = 6 per group). **E** Western blot assays of protein expression levels of NF-κB P65 within the DG region (N = 6 per group). *P < 0.05, ***P < 0.001, LPS vs Control group; ^#^*P* < 0.05, ^###^*P* < 0.001, LPS vs LPS + AGO (AGO, Agomelatine). Data are presented as means ± SEMs

Typical pathological changes in response to LPS consist of an increase in neuroinflammation. The results from our immunofluorescent assays revealed that the number of Iba-1 + microglia within the DG region was significantly decreased in LPS rats receiving agomelatine (*F*_(3,15)_ = 6.090, *P* < 0.01, Fig. 3A). As glial cell activation usually triggers secretions of cytokines, we next assessed expression levels of several critical pro-inflammatory cytokines and NF-κB P65. In response to agomelatine treatment, mRNA expression levels of the pro-inflammatory cytokines TNF-α (*F*_(3,15)_ = 8.934, *P* < 0.01), IFN-γ (*F*_(3,15)_ = 20.13, *P* < 0.0001) and IL-1β (*F*_(3,15)_ = 20.56, *P* < 0.0001) and protein levels of NF-κB (*F*_(3,15)_ = 8.717, *P* < 0.01), within the DG area of LPS rats were all significantly decreased as compared to that observed in rats receiving LPS alone (Fig. 3B–E). Therefore, these results suggest that agomelatine reduces neuroinflammatory responses in the DG region of these LPS-induced depressed rats.

### Agomelatine decreases the enhanced autophagy and synaptic damage in the DG region of LPS rats

**Fig. 4:**
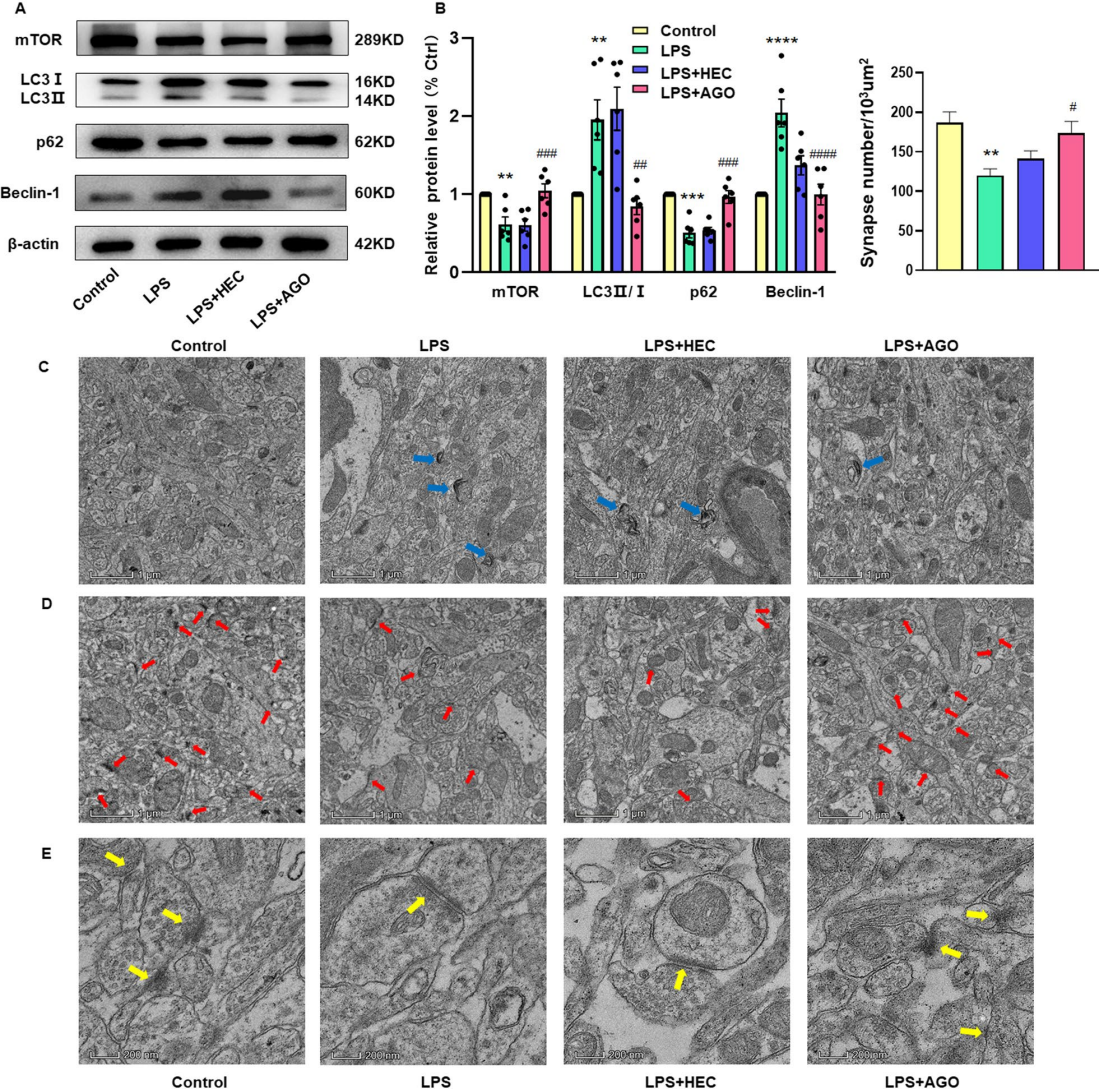
Agomelatine decreased the enhanced autophagy and synaptic damage in the DG region of LPS rats. **A**, **B** Western blot assays of protein expression levels of autophagy-related proteins within the DG region (*N* = 6 per group). **C** Representative electronic micrographs and summary of data showing the number of autolysosomes/cell. Arrows indicate autolysosomes. Scale bar is 1 μm. (D) Representative electron micrographs and summary of data showing the number of synapsis. Arrows indicate neuronal synapses. Scale bar is 1 μm (*N* = 6 per group). **E** Representative electron micrograph of DG neurons in rats from each group (*N* = 6 per group). Arrows indicate neuronal synapses. Scale bar is 0.2 μm.*P < 0.05, **P < 0.01, ***P < 0.001, ****P < 0.0001, LPS vs Control group; ^#^*P* < 0.05, ^##^*P* < 0.01, ^###^*P* < 0.001, ^####^*P* < 0.001, LPS vs LPS + AGO (AGO, Agomelatine). Data are presented as means ± SEMs

As excessive inflammation can lead to dysregulation of autophagy, we examined the effects of agomelatine on autophagy in the hippocampal DG region of LPS rats. When compared with the control group, the autophagy-related proteins, LC3II/I and Beclin-1, were significantly increased, while expression levels of proteins that inhibit autophagy were decreased in LPS rats. The depression associated with LPS treatment promoted autophagic processes in the DG area, while agomelatine treatment reversed this enhanced autophagy (mTOR: *F*_(3,15)_ = 18.62, *P* < 0.0001, LC3II/I: *F*_(3,15)_ = 12.73, *P* < 0.001, P62: *F*_(3,15)_ = 28.26, *P* < 0.0001, Beclin-1: *F*_(3,15)_ = 22.95, *P* < 0.0001, Fig. 4A, B). The results from TEM also showed that agomelatine reduced the number of typical autolysosomes in neurons within the DG region (Fig. 4C). As deregulation of the autophagy pathway produces neuronal and synaptic plasticity loss as well as hippocampal shrinkage and LPS contributes to this dysregulation, the significant reductions in bouton and mushroom spine volume as well as the decreased post-synaptic density (PSD) in these synapses following LPS are explicable [30]. Notably, agomelatine improved this synaptic damage and increased synapse numbers in these LPS rats (synapse numbers: *F*_(3,15)_ = 6.263, *P* < 0.01, Fig. 4D, E).

### Agomelatine reduces neuronal apoptosis in the DG region of LPS rats

**Fig. 5:**
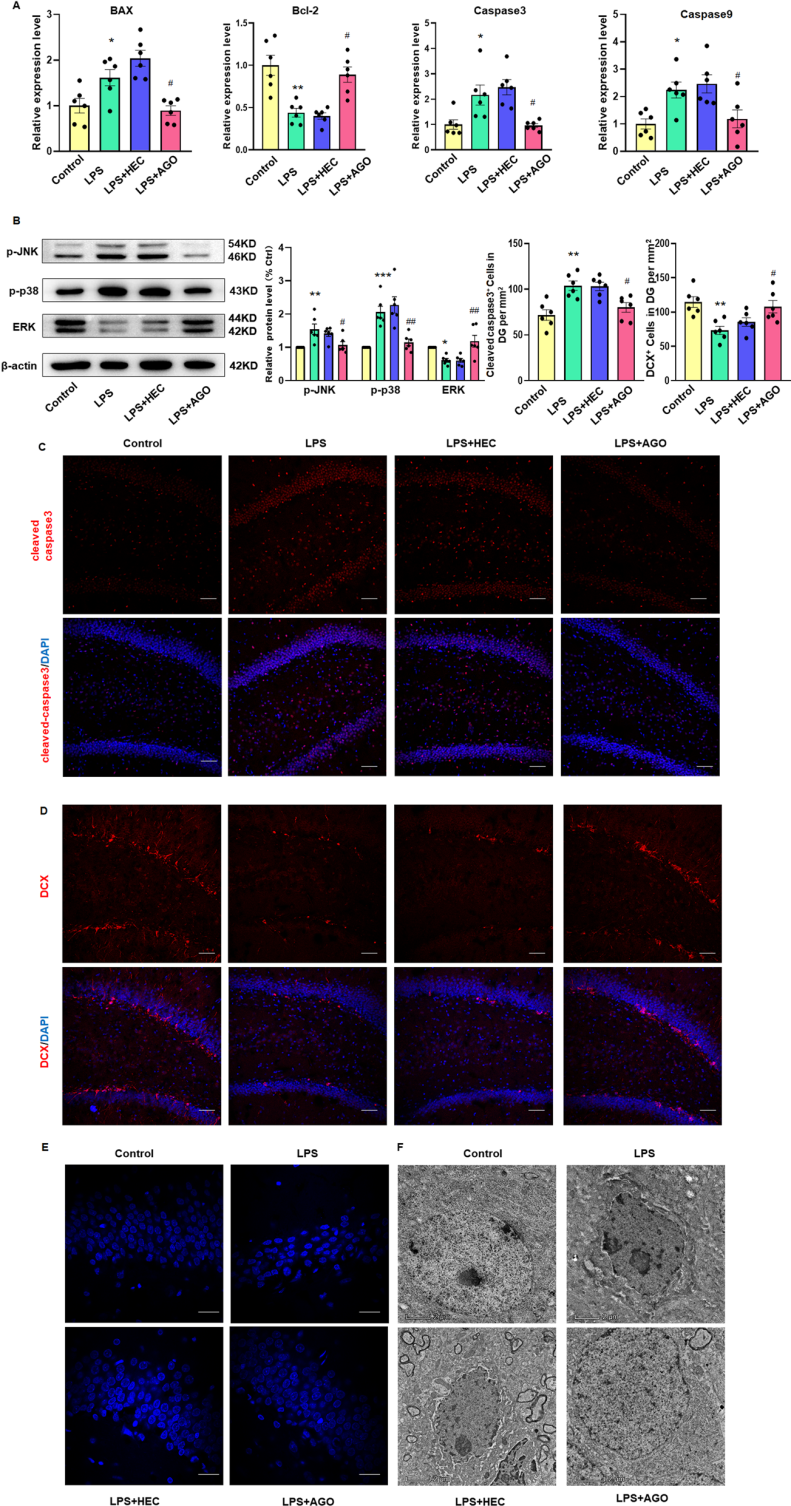
Agomelatine reduces neuronal apoptosis within the DG region of LPS rats. **A** Q-PCR assays of mRNA expression levels of Bcl-2, Bax, Caspase3 and Caspase9 within the DG region. Band intensities were normalized to GAPDH (*N* = 6 per group). **B** Western blot assays of protein expression levels of ERK, p-p38 and p-JNK within the DG region (*N* = 6 per group). **C** Representative images of cleaved-Caspase3 (red). Nuclei (blue) are stained with DAPI. Scale bar is 50 μm (*N* = 6 per group). **D** Representative images of DCX (red). Scale bar is 50 μm (*N* = 6 per group). **E** Representative images of Hoechst-33258 staining (Scale bar is 5 μm). **F** TEM showing morphological changes of nuclei (Scale bar is 2 μm) *P < 0.05, **P < 0.01, ***P < 0.001, LPS vs Control group; ^#^*P* < 0.05, ^##^*P* < 0.01, ^###^*P* < 0.001, LPS vs LPS + AGO (AGO, Agomelatine). Data are presented as means SEMs

We have proposed that agomelatine inhibits ASK1 activation by antagonizing the 5-HT2C receptor, and thus exerts a neuroprotective effect against depression. To test this hypothesis, we examined the potential for anti-apoptotic effects of agomelatine. Transcription levels of the apoptosis-related proteins Bax (*F*_(3,15)_ = 13.38, *P* < 0.001), caspase 3 (*F*_(3,15)_ = 8.228, *P* < 0.01) and caspase-9 (*F*_(3,15)_ = 9.662, *P* < 0.001) were all significantly increased in the DG region of LPS rats, while expression levels of Bcl-2 (*F*_(3,15)_ = 12.67, *P* < 0.0001) were decreased. All these changes were reversed with agomelatine treatment (Fig. 5A). As shown in Fig. 5B, agomelatine increased the expression of ERK, which is related to cell proliferation, while expressions of the apoptosis-related molecules, JNK and P38, were decreased (p-JNK: *F*_(3,15)_ = 8.481, *P* < 0.01, p-p38: *F*_(3,15)_ = 19.96, *P* < 0.0001, ERK: *F*_(3,15)_ = 10.27, *P* < 0.001). The results from our immunofluorescent assay showed that cleaved-Caspase3 was decreased (F _(3, 15)_ = 9.300, *P* < 0.01, Fig. 5C) after agomelatine treatment, indicative of a reduction in neuronal apoptosis, while the increase of DCX suggested that this agomelatine treatment promoted neuronal regeneration (*F*_(3,15)_ = 6.743, *P* < 0.01, Fig. 5D). We also examined the morphology of nuclei within these neurons as viewed with use of electron (Fig. 5F) and fluorescent (Fig. 5E) microscopy. Agomelatine improved the aggregation and marginal morphology of nuclear chromatin within these DG neurons. In this way, agomelatine may decrease the activation of ASK1 and downstream JNK P38 through antagonism of the 5-HT2C receptor, to produce its antidepressant effects.

## Discussion

Depression is a complex neuropsychiatric disorder characterized by behavioral, cognitive and emotional changes [31, 32]. It is most often associated with depressed or flat moods, reduced interest in novel events, increased despair, changes in sleep patterns and the presence of suicidal thoughts [33]. The complex nature of this disorder indicates the difficulty, but need, for more effective and well-tolerated antidepressants. Although it represents a relatively new antidepressant drug, agomelatine has been approved for use in the clinical treatment of depression [34]. Agomelatine has been shown to exert a variety of effects. For example, it can act on melatonin (MT1 and MT2) and 5-HT2C receptors. According to findings from previous reports, agomelatine has been shown to promote dopamine production in the prefrontal cortex and increase cell proliferation in the hippocampus of rats, as well as promote neuronal growth, cell survival and increase the level of circulating BDNF [35–37]. Owing to its antioxidant and anti-inflammatory properties, agomelatine has also been shown to exert a number of effects in other tissues and systems, such as the cardiovascular system, liver and kidney [38–40] and results from in vitro experiments have shown that agomelatine can reduce oxidative stress by inhibiting ROS production [41]. Additional effects of agomelatine include protection of myocardial ischemia reperfusion injury by enhancing GSK-3β phosphorylation [42] and repair of the blood−brain barrier through inhibiting the infiltration of macrophages into brain tissue [43]. Although the findings from these reports reveal a number of treatment effects exerted by agomelatine in different diseases, at present, the potential molecular mechanisms as related to its neuroprotective effects as a neutral antagonist of 5-HT2C receptor in regulating neuronal intracellular signaling have yet to be established.

In this study, we first demonstrated that agomelatine (40 mg/kg) can improve anxiety and depression-like behaviors in an LPS-induced rat model of depression. The 5-HT2C receptor, which is the most widely distributed receptor in the central nervous system, can regulate 5-HT activity in almost all regions of the brain [44, 45] and represents an important target for the treatment of emotional, cognitive and metabolic disorders as well as drug addiction. Activation of the 5-HT2C receptor induces anxiety [46] and 5-HT2C receptor knockout mice show a reduction in anxiety-like behavior [47]. Currently, a number of antidepressant drugs, such as imipramine, amitriptyline, amoxapine and clomipramine, have all been shown to function as antagonists of the 5-HT2C receptor [44, 48]. Given this background information, we examined the potential for agomelatine to serve as a 5-HT2C receptor antagonist, as one of the possible molecular mechanisms through which it could function as an antidepressant. To accomplish this goal, we first confirmed that 5-HT2C receptor expression was present in rat hippocampal neurons within the DG region. It has been reported that inhibitory G protein-coupled receptors play a key role in regulating the central and peripheral nervous systems [49, 50]. Gαi-2 was shown to block the transient inhibition of mGluR2 on synaptic transmission and enhances LTD [51]. In the present study, results from our immunofluorescent co-localization and immunoprecipitation assays, revealed that Gαi-2 was coupled with the 5-HT2C receptor and agomelatine can inhibit Gαi-2 conjugated with the 5-HT2C receptor. As a result, there is an increase in the expression of cAMP and activation of cyclic-AMP-dependent protein kinase A (PKA) as achieved through antagonism of 5-HT2C receptors. As the activation of PKA further activates TRX-1, thus inhibiting the expression of ASK1 [52–55], we infer that agomelatine can play a neuroprotective role in the down-stream intracellular cAMP-PKA-ASK1 signal pathway through the coupling of 5-HT2C receptors and Gαi-2. Functionally coupled between 5-HT2c receptor and Gαi/o has previously been described in Xenopus oocytes. [56, 57], we present evidence that this mechanism may be one of the important reasons for the antidepressant effects of agomelatine.

The results from recent studies have provided evidence indicating that neuroinflammatory processes may play a key role in the pathogenesis of depression and other central nervous system disease [58–60]. Such inflammation combined with autophagy can interact in the pathogenesis of depression. Autophagy is a protective process that monitors the metabolic needs of cells and renewal of certain organelles [61]. In pathological conditions of abnormal autophagy there is an increase in the number of damaged mitochondria, which can then trigger cell apoptosis through internal pathways and ROS production to destabilize lysosome membranes, thus aggravating neuroinflammation [11, 62]. Here, we demonstrate that neuroinflammation, abnormal autophagy and neuronal apoptosis within the hippocampal DG region of rats were significantly increased after LPS injection. The capacity for agomelatine to diminish these effects as well as play a neuroprotective role in the hippocampal DG region, reveals some of the potential molecular mechanisms for the beneficial effects of agomelatine in the treatment of depression.

It should be pointed out that this study mainly focused on exploring the regulatory effects of agomelatine which act as a 5-HT2C receptor neutral antagonist on behavior and neurobiological changes in the hippocampal DG region of LPS-induced depression rat model. The results of this study showed that agomelatine significantly suppressed neuroinflammation, abnormal autophagy and neuronal apoptosis in the hippocampal DG region by inhibiting 5-HT2C receptor conjugated with Gαi-2, and then activating intracellular PKA and inhibiting ASK1 signaling pathway. However, agomelatine is also a melatonin receptor agonist. Some researchers have proposed that MT1 and MT2 can physically associate with 5-HT2C receptors and form heteromers when expressed in transfected HEK293 cells [63]. The existence of this structure also may be the reason for the antidepressant efficacy of agomelatine Therefore, whether agomelatine involved in the regulation of neuroprotective as a melatonin receptor agonist, and its potential related mechanisms are also worthy of further investigation. Second, the purpose of this study was to induce depression-like behavior and increased neuroinflammation in rats by LPS injection, we used LPS injection for 10 days to investigate the neuroprotective effect of agomelatine on inflammation-related depression, which consistent with what we have observed in this study. Finally, the hippocampus is the most commonly studied brain region in depression research, previous literature suggests that, the hippocampus, in particular the DG region, is thought to be involved in mood and cognitive regulation, undergoes severe damage during the onset of depression, and has been shown the smaller volume in the DG region in depression-like animals and in patients with depression in clinical trials [64, 65]. Therefore, we chose to focus on the possible mechanism of neuroprotection of DG region by agomelatine. Depression is a complex disease that involves potential interactions between gene−environment and gene−sex factors, and the 5-HT2C receptor has been reported to widely distributed in the central nervous system [66], so it is reasonable to speculate that the protective effects of agomelatine may be present in multiple brain regions associated with depression. In the animal model of depression used in this study, we observed that agomelatine plays a good neuroprotective role in the hippocampal DG region at the cellular and molecular levels. However, the detailed mechanisms of agomelatine in regulating neuronal function in antidepressant treatment remain to be further explored.

## Conclusion

In conclusion, the findings of this study provide some new insights into the possible molecular mechanisms for the antidepressant effects of agomelatine. Specifically, we propose that agomelatine activates intracellular PKA and inhibits ASK1 expression through coupling of the 5-HT2C receptor with Gαi-2. This antidepressant capacity appears to, at least in part, be achieved by inhibiting excessive autophagy and apoptosis of cells and exerting a neuroprotective effect. Although our data provide relatively strong support for these hypothesized mechanisms, further research will be required to corroborate these findings. It is also important to note that these molecular mechanisms, as mediated by 5-HT2C receptors coupled with Gαi-2 may also be involved in the pathological process of other central nervous system-related diseases and may also represent possible mechanisms for the effects of other 5-HT2C receptor antagonists as related to antidepressant effects. Taken together, the results of this study reveal a new potential mechanism for the antidepressant effects of agomelatine and provide the basis for the development of novel strategies in the treatment of depression.

## Supplementary Information

**Additional file 1: Figure S1.** The protein expression of 5-HT2C receptor on the cell membrane. (A)) Western blot movementassays of protein expression levels of 5-HT2C receptor on the cell membranewithin the DG region (N = 6 per group). NS P > 0.05 LPS vs Control group; LPS vs LPS + AGO (AGO, Agomelatine). Data are presented as means SEMs.

**Additional file 2: Figure S2.** Effects of agomelatine on autonomous movement in rats. (A) Schematic diagram of movement paths. (B) Agomelatine reversed the decreases in the time spent in the central area in the OFT. (C) The significant difference in total distance was not observed in any of the groups. N = 6 per group, **P < 0.01, LPS vs Control group; ^#^*P* < 0.05, LPS vs LPS + AGO (AGO, Agomelatine). Data are presented as means ± SEMs.

**Additional file 3: Figure S3.** Agomelatine has no effects on behavioral and 5-HT2C signaling pathway in rats. (A) Agameratine had no effect on percent of sucrose consumption with normal rats in the SPT and (B) didn’t increased immobility time in normal rats. (C) The significant difference in the time spent and (D) the total distance was not observed in two groups. (E) Western blot assays of protein expression levels of the 5-HT2C receptor and P-PKA within the DG region. N = 5 per group, ^NS^P > 0.05, Control + AGO (AGO, Agomelatine) vs Control group; Data are presented as means ± SEMs.

**Additional file 4: Table S1.** Primer sequences of target genes used for Reverse transcription PCR in this study.

## Data Availability

The data that support the findings of this study are available from the corresponding author upon reasonable request.

## Abbreviations

LPS: Lipopolysaccharide
DG: Dentate gyrus
Gαi-2: G alphai (2)
ASK1: Apoptosis signal-regulating kinase 1
MPKA: Mitogen-activated protein kinase
ROS: Reactive oxygen species
BDNF: Brain-derived neurotrophic factor
HEC: Hydroxyethyl cellulose
SPT: Sucrose preference test
FST: Forced swimming test
EPM: Elevated plus maze
PFA: Paraformaldehyde
TEM: Transmission electron microscopy
ELISA: Enzyme-linked immunosorbent assay
cAMP: Cyclic adenosine monophosphate
ANOVA: Analysis of variance
PSD: Post-synaptic density
PKA: Cyclic-AMP-dependent protein kinase A
