# Supplementary figures and images for: Agomelatine rescues lipopolysaccharide-induced neural injury and depression-like behaviors via suppression of the Gαi-2-PKA-ASK1 signaling pathway
Source: J Neuroinflammation. 2022 May 24;19:117. doi: 10.1186/s12974-022-02479-x (PMC9131561; doi:10.1186/s12974-022-02479-x)

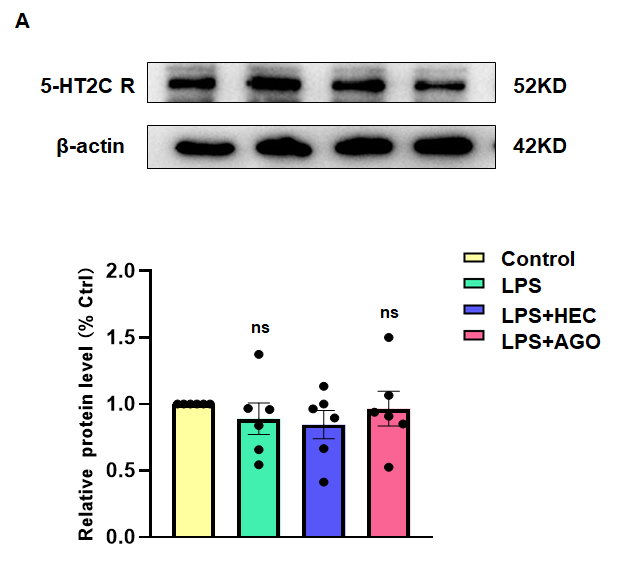

Supplement: Supplementary file 1 — Additional file 1: Figure S1. The protein expression of 5-HT2C receptor on the cell membrane. (A)) Western blot movementassays of protein expression levels of 5-HT2C receptor on the cell membranewithin the DG region (N = 6 per group). NS P > 0.05 LPS vs Control group; LPS vs LPS + AGO (AGO, Agomelatine). Data are presented as means SEMs. [file 12974_2022_2479_MOESM1_ESM.tif]

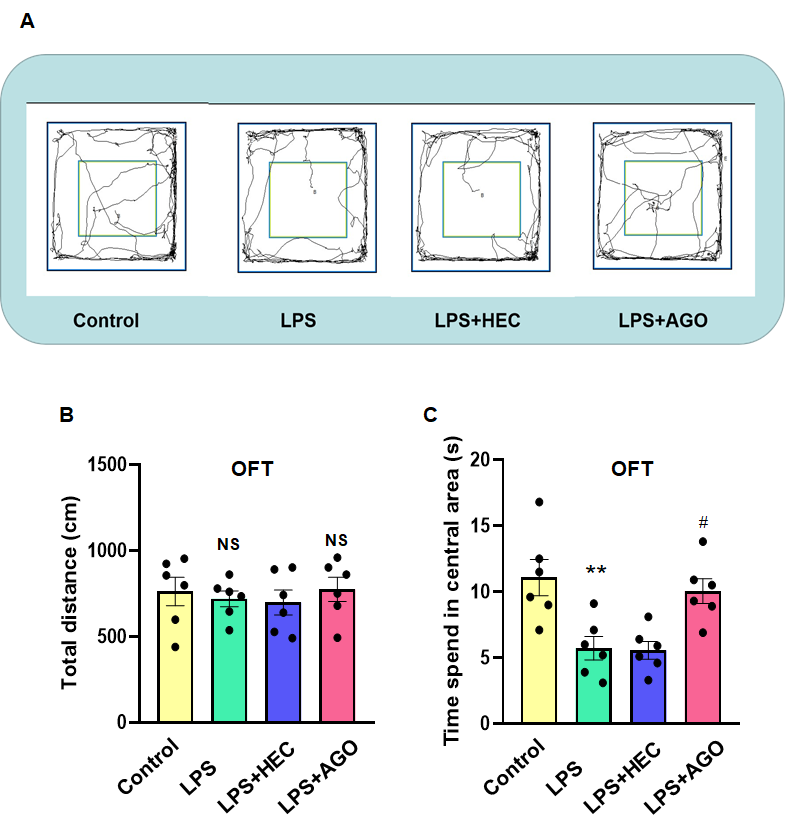

Supplement: Supplementary file 2 — Additional file 2: Figure S2. Effects of agomelatine on autonomous movement in rats. (A) Schematic diagram of movement paths. (B) Agomelatine reversed the decreases in the time spent in the central area in the OFT. (C) The significant difference in total distance was not observed in any of the groups. N = 6 per group, **P < 0.01, LPS vs Control group; #P < 0.05, LPS vs LPS + AGO (AGO, Agomelatine). Data are presented as means ± SEMs. [file 12974_2022_2479_MOESM2_ESM.tif]

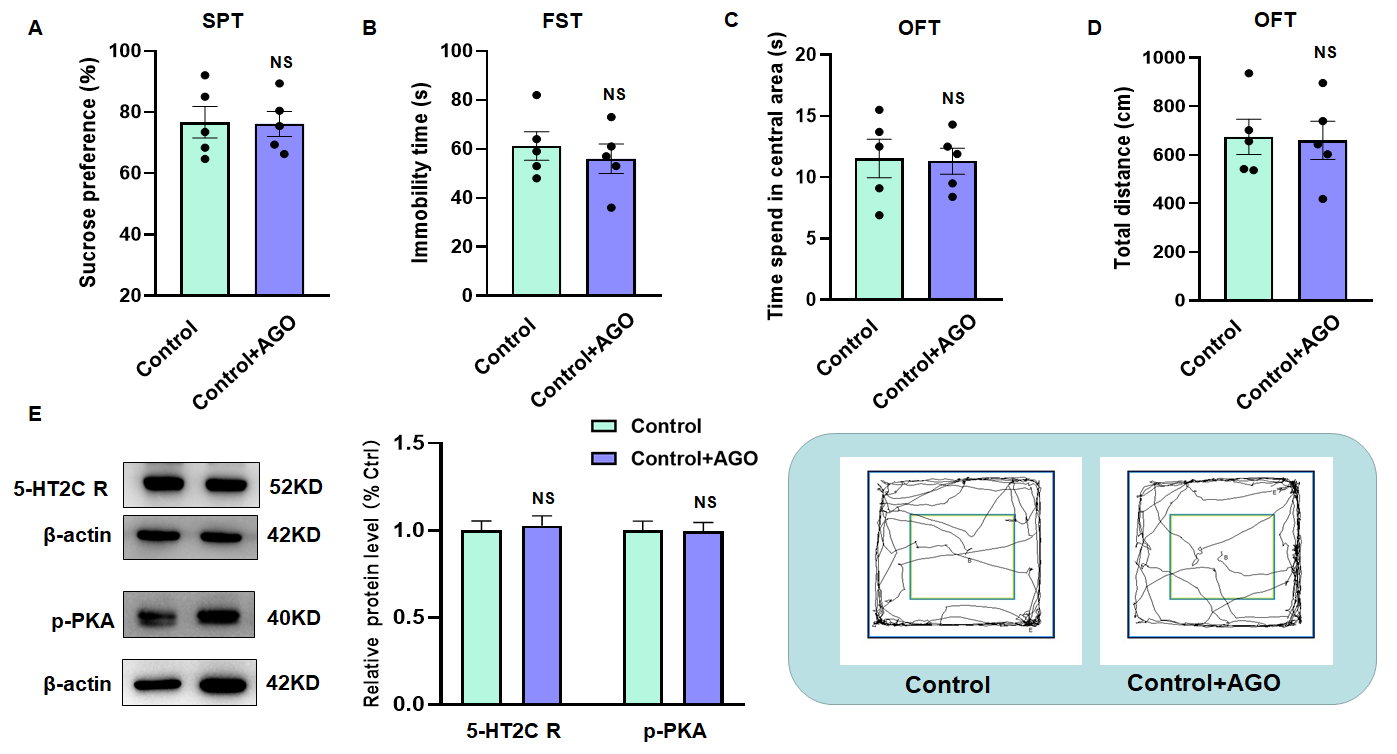

Supplement: Supplementary file 3 — Additional file 3: Figure S3. Agomelatine has no effects on behavioral and 5-HT2C signaling pathway in rats. (A) Agameratine had no effect on percent of sucrose consumption with normal rats in the SPT and (B) didn’t increased immobility time in normal rats. (C) The significant difference in the time spent and (D) the total distance was not observed in two groups. (E) Western blot assays of protein expression levels of the 5-HT2C receptor and P-PKA within the DG region. N = 5 per group, NSP > 0.05, Control + AGO (AGO, Agomelatine) vs Control group; Data are presented as means ± SEMs. [file 12974_2022_2479_MOESM3_ESM.tif]
