# Supplementary material for: Agomelatine rescues lipopolysaccharide-induced neural injury and depression-like behaviors via suppression of the Gαi-2-PKA-ASK1 signaling pathway
Source: J Neuroinflammation. 2022 May 24;19:117. doi: 10.1186/s12974-022-02479-x (PMC9131561; doi:10.1186/s12974-022-02479-x)
Supplement: Supplementary file 4 — Additional file 4: Table S1. Primer sequences of target genes used for Reverse transcription PCR in this study. [file 12974_2022_2479_MOESM4_ESM.docx]

Supplementary Table S1. Primer sequences of target genes used for Reverse transcription PCR in this study

| Gene | Forword (5’→3’) | Reverse (5’→3’) |
| --- | --- | --- |
| IL-1β | AAG ATG AAG GGC TGC TTC CAA ACC | ATA CTG CCT GCC TGA AGC TCT TGT |
| IFN-γ | ATT CAT GAG CAT CGC CAA GTT C | TGA CAG CTG GTG AAT CAC TCT GAT |
| TNF-α | TGA TCG GTC CCA ACA AGG A | TGC TTG GTG GTT TGC TAC GA |
| Bcl-2 | GGA TCC AGG ATA ACG GAG GC | ATG CAC CCA GAG TGA TGC AG |
| Bax | TCT TCA AAC TGC TGG GCC ATT | CTT GTC ACC TGC CTG ACT GCT |
| Caspase3 | GGA GCT TGG AAC GCG AAG AA | ACA CAA GCC CAT TTC AGG GT |
| Caspase9 | CAA GAA GAG CGG TTC CTG GT | CAG AAA CAG CAT TGG CGA CC |
| GAPDH | AGT GCC AGC CTC GTC TCA TA | GGT AAC CAG GCG TCC GAT AC |
